# *Beta-2-microglobulin* Mutations Are Linked to a Distinct Metastatic Pattern and a Favorable Outcome in Microsatellite-Unstable Stage IV Gastrointestinal Cancers

**DOI:** 10.3389/fonc.2021.669774

**Published:** 2021-06-08

**Authors:** Elena Busch, Aysel Ahadova, Kosima Kosmalla, Lena Bohaumilitzky, Pauline L. Pfuderer, Alexej Ballhausen, Johannes Witt, Jan-Niklas Wittemann, Hendrik Bläker, Elke Holinski-Feder, Dirk Jäger, Magnus von Knebel Doeberitz, Georg Martin Haag, Matthias Kloor

**Affiliations:** ^1^ Department of Medical Oncology, National Centre for Tumor Diseases, Heidelberg University Hospital, Heidelberg, Germany; ^2^ Department of Applied Tumor Biology, Heidelberg University Hospital, Clinical Cooperation Unit Applied Tumor Biology, German Cancer Research Centre (DKFZ), Heidelberg, Germany; ^3^ Institute of Pathology, University Hospital Leipzig, Leipzig, Germany; ^4^ Medizinische Klinik und Poliklinik IV, Klinikum der Universität München, Munich, Germany; ^5^ MGZ – Medical Genetics Centre, Munich, Germany

**Keywords:** MSI cancer, metastatic pattern, immune checkpoint blockade, *B2M* mutation, prognosis, therapy response

## Abstract

Immune checkpoint blockade (ICB) shows remarkable clinical effects in patients with metastatic microsatellite-unstable (MSI) cancer. However, markers identifying potential non-responders are missing. We examined the prevalence of *Beta-2-microglobulin* (*B2M)* mutations, a common immune evasion mechanism, in stage IV MSI gastrointestinal cancer and its influence on metastatic pattern and patients’ survival under ICB. Twenty-five patients with metastatic, MSI gastrointestinal adenocarcinoma were included. Eighteen patients received ICB with pembrolizumab and one patient with nivolumab/ipilimumab. Sequencing was performed to determine *B2M* mutation status. *B2M* mutations and loss of B2M expression were detected in 6 out of 25 stage IV MSI cancers. *B2M* mutations were strongly associated with exclusively peritoneal/peritoneal and lymph node metastases (p=0.0055). However, no significant differences in therapy response (25% *vs*. 46.6%, p>0.99) and survival (median PFS: 19.5 *vs* 33.0 months, p=0.74; median OS 39 months *vs*. not reached, p>0.99) were observed between *B2M*-mutant and *B2M*-wild type tumor patients. Among metastatic MSI GI cancers, *B2M*-mutant tumors represent a biologically distinct disease with distinct metastatic patterns. To assess ICB response in *B2M*-mutant MSI cancer patients, future studies need to account for the fact that baseline survival of patients with *B2M*-mutant MSI cancer may be longer than of patients with *B2M*-wild type MSI cancer.

## Introduction

DNA mismatch repair (MMR) deficiency is one of the major mechanisms enabling genomic instability in cancer. MMR-deficient cancers accumulate a high load of somatic mutations, predominantly insertion/deletion mutations at microsatellite sequences (microsatellite instability, MSI). Insertion/deletion mutations of coding microsatellites can cause shifts of the translational reading frame and generation of long *neo*antigen stretches containing *neo*epitopes (1). These *neo*antigens elicit strong immune responses against MSI tumors (2).

However, exhaustion can limit the efficacy of T cell responses upon prolonged antigen exposure without elimination of target cells due to the binding of inhibitory receptors, such as PD-1 (programmed cell death 1), to one of its ligands, most prominently PD-L1 (3). Blockade of these immune checkpoints with re-activation of T cells has therefore had remarkable clinical success particularly in MSI cancer patients (4).

Although immune checkpoint blockade (ICB) can lead to complete responses in a subset of patients, a substantial proportion of MSI cancer patients do not respond to ICB, and predictors of therapy response among MSI cancer patients are lacking.

Besides misdiagnosis of MMR-proficient tumors as MSI (5), non-functionality of tumor cells’ antigen presentation machinery, mediated by mutations of the *Beta2-microglobulin* (*B2M*) gene, has been discussed as a key mechanism of resistance towards ICB in some cancer types, such as melanoma (6, 7).

MSI cancers commonly present with *B2M* mutations as a mechanism of immune evasion (8, 9). In contrast to other tumor types including melanoma or non-small cell lung cancer, MSI colorectal cancers display biallelic *B2M* mutations in up to 30% already at the time point of diagnosis (8). An association of *B2M* mutations with improved survival under adjuvant therapy had previously been reported for MSI colorectal cancer patients (10).

We therefore asked whether the response to immunotherapy and the metastatic pattern of stage IV MSI cancers at the time point of diagnosis may differ depending on *B2M* mutation status.

## Methods

### Material Collection

Clinical data and tumor material (formalin-fixed, paraffin-embedded (FFPE) tumor specimens) from patients with metastatic MSI GI cancer were collected at the National Center for Tumor Diseases, Heidelberg, Germany. The study was performed in accordance with the Declaration of Helsinki. Written informed consent was obtained from all patients and the study was approved by the Ethics Committee (V5.1 S207/2005).

### Molecular Tumor Testing

5 µm FFPE tissue sections were stained using hematoxylin&eosin, histologically analyzed and manually microdissected to obtain DNA. MSI status was determined using a combination of three mononucleotide markers (BAT25, BAT26, CAT25) and three dinucleotide markers (D2S123, D5S346, D17S250) as described previously (11). *B2M* mutation status was determined using targeted sequencing, as previously (12). Briefly, PCR amplification of B2M exons 1 and 2 was performed using primer sequences: Exon 1 For— GGCATTCCTGAAGCTGACA, Exon 1 Rev— AGAGCGGGAGAGGAA GGAC, Exon 2a For—TTTCCCGATATTCCTCAGGTA, Exon 2a Rev— AATTCAGTGTAGTACAAGAG and Exon 2b For—TGTCTTTCA GCAAGGACTGG, Exon 2b Rev—CAAAGTCACATGGTTCACACG. The obtained PCR products (QIAquick PCR Purification Kit) were purified, and the sequencing reaction was performed using the BigDye^®^ Terminator v1.1 Cycle Sequencing Kit (Thermo Fisher Scientific, Wilmington, DE, USA). After dissolving the precipitated products in 12 μl of HiDi Formamide (Thermo Fisher Scientific, Wilmington, DE, USA), sequencing was performed on ABI 3130*xl* Genetic Analyser and analyzed using Sequencing Analysis Software V6.0 (Applied Biosystems). B2M protein expression was analyzed by immunohistochemistry staining using a standard protocol described before (13).

### Statistical Analysis

Statistical analyses for categorical data were performed using two-tailed Fisher’s exact test. Kaplan-Meier curves for progression-free survival (PFS) and overall survival (OS) were generated using GraphPad Prism (Version 6). Statistical significance was analyzed using log-rank test. A logistic regression model was estimated using the glm (generalized linear model) function within the stats package (14). R: A language and environment for statistical computing. R Foundation for Statistical Computing, Vienna, Austria. URL https://www.R-project.org/). To predict the binary outcome (peritoneal/lymphatic metastasis or non- peritoneal/lymphatic metastasis) we use *B2M* mutation status and the site of the primary tumor (colorectal or gastric cancer, excluded cholangiocellular carcinoma) as predictors.

## Results

### Patient Characteristics

Primary tumors (19 colorectal, 5 gastric, and 1 cholangiocellular carcinomas) from 25 patients (13 female and 12 male, median age 65 years (range 29 to 78) presenting with a stage IV MSI adenocarcinoma have been analyzed in this study. The clinical and molecular data are summarized in **Table 1**.

**Table 1:** Clinical and molecular patient characteristics.

Patient	Age	Gender	Diagnosis	Metastic site	B2M satatus	ICB therapy	Therapy line	Best response
1	29	female	CCC	HEP, PUL	wt	p	3	CR
2	64	male	CRC, sigma	OTH (local recurrence)	wt	p	1	n.a.
3	53	male	CRC, transversum	HEP	wt	p	3	PD
4	68	female	CRC, sigma	PER	wt	p	1	PD
5	65	male	CRC, ascendens	PER, ADR, OTH (spleen), LYM	wt	p	2	PD
6	47	male	CRC, transversum	PUL, HEP, LYM, PER	wt	p	3	PR
7	42	female	CRC, cecum	HEP, PUL, PER	wt	p	3	PR
8	34	female	CRC, transversum	HEP	wt	p	2	PR
9	67	male	CRC, RF	HEP, PER	wt	p	1	PR
10	77	female	CRC, ascendens	LYM, OTH (kidney)	wt	p	2	PR
11	72	female	Crc, rectum	HEP	wt	p	2	PR
12	41	male	CRC, ascendens	HEP, PER, LYM, OTH (muscle)	wt	p	7	SD
13	73	female	CRC, transversum	PUL, HEP, PER	wt	p	2	SD
14	44	female	CRC, cecum	OSS, OTH (ovary)	wt	p	2	SD
15	62	female	CRC, transversum	HEP, LYM, PER	wt	p	2	SD
16	54	female	CRC, cecum	LYM, PER, OTH (ovary, uterus)	wt	(-)	(-)	(-)
17	67	male	gastric cancer	BRA (resected and irradiated)	wt	(-)	(-)	(-)
18	54	male	gastric cancer	ADR (irradiated)	wt	(-)	(-)	(-)
19	78	male	gastric cancer	ADR, LYM (irradiated)	wt	(-)	(-)	(-)
20	36	male	CRC, cecum	PER (resected)	mut	(-)	(-)	(-)
21	71	male	CRC, descendens	PER, LYM	mut	p	1	PR
22	52	male	CRC, LF	PER, LYM, HEP	mut	p	3	SD
23	70	female	gastric cancer	PER, LYM	mut	p	2	SD
24	38	female	gastric cancer	PER, OSS, MAR,ADR	mut	n/i	2	SD
25	69	female	CRC,cecum	PER (resected)	mut	(-)	(-)	(-)

18 out of 19 patients received pembrolizumab (p), one gastric cancer patient (#24) was treated with nivolumab first and, failing to respond, was then escalated to nivolumab/ipilimumab (n/i) combination therapy. CRC, colorectal cancer; CCC, cholangiocellular carcinoma; LF, left flexure; RF, right flexure.

### Metastatic Patterns According to *B2M* Status

Previously, *B2M* mutations had been shown to be associated with locally restricted disease in MSI GI cancer patients (8, 10). In our study, however, 6 out of 25 (24%) tumors presented with biallelic *B2M* mutations (4 colorectal, 2 gastric cancers, **Table 1**), demonstrating that *B2M* mutations can also occur in stage IV MSI GI cancers. Immunohistochemical staining revealed loss of B2M protein expression in all *B2M*-mutant tumors, supporting biallelic inactivation and loss of function.

Interestingly, metastatic patterns differed between patients with *B2M*-mutant and *B2M*-wild type tumors. *B2M*-mutant tumor patients predominantly presented with peritoneal metastases, with 2 patients having exclusively peritoneal and 2 patients with peritoneal and lymph node metastases, in contrast to only one patient with a singular hepatic metastasis. On the contrary, only one out of 19 patients with *B2M*-wild type tumors had an exclusively peritoneal/peritoneal and lymph node metastatic pattern (p=0.0055), but 10/19 patients showed extensive hepatic disease (**Table 1**).

As the analyzed patient cohort was heterogeneous with regard to the primary tumor site, which could also influence the observed metastatic pattern, we estimated a logistic regression model using the GLM (generalized linear model) function. Mutant *B2M* status (vs wt *B2M*) increased the log odds of peritoneal/peritoneal and lymphatic metastasis (vs non-peritoneal/peritoneal and lymphatic metastasis) by 3.873 (p=0.00968). The coefficients for primary tumor location (colorectal or gastric cancer) had no significant effect on the metastasis location (log odds -1.283, p value 0.45063).

We also calculated the average marginal effect (AME) for both predictors (*B2M* status and primary site of the tumor). The AME of mutant *B2M* status on metastasis location is 0.6494 (~65%), meaning that on average a *B2M*-mutant tumor has a ~65% higher probability of having a peritoneal/peritoneal and lymphatic metastasis (p = 0.0006). The primary tumor site had no significant AME on the metastatic pattern (p value 0.3952, AME = -0.1063).

### Response to ICB Therapy

**Figure 1:**
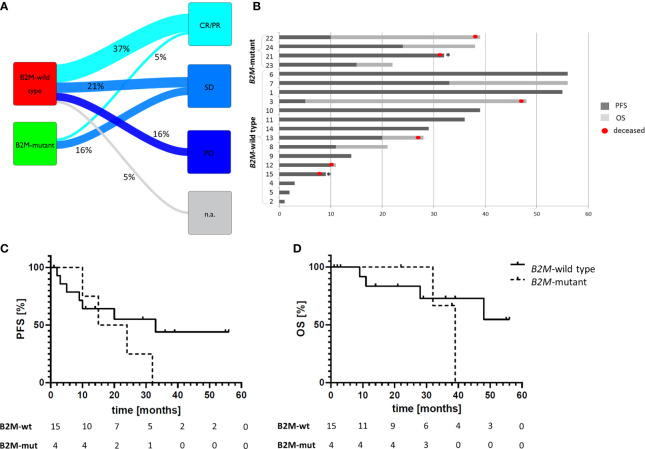
ICB therapy response depending on the tumor *B2M* mutation status**. (A)** Sankey diagram summarizing the therapy responses in 19 MSI GI cancer patients under ICB therapy. **(B)** Swimmer plot describing progression free survival (dark grey) and follow-up duration in 19 patients. Deceased patients are marked with a red dot, death unrelated to tumor disease is marked with an asterisk (*): one patient died because of concurrent cardiovascular disease, one patient died in septic shock, both patients did not show any signs of disease progression at this point. Patients #6-11, and 21 demonstrated PR, and patient #1 demonstrated CR as best response. Numbers refer to Patient IDs in **Table 1**. **(C, D)** Survival curves of patients receiving ICB therapy depending on the *B2M* status of the tumor: progression-free **(C)** and overall survival **(D)**. Despite nominal differences in PFS of *B2M*-mutant (n=4) and *B2M*-wild type (n=15) tumor patients (median PFS: 19.5 *vs* 33.0 months, respectively, p=0.74), the OS did not differ between these two patient groups (median OS 39 months *vs*. not reached, respectively, p>0.99), indicating good prognosis of patients with *B2M*-mutant tumors irrespectively of immune checkpoint blockade therapy. Statistical significance was analyzed using log-rank test.

Nineteen out of 25 patients received ICB therapy with pembrolizumab or nivolumab/ipilimumab (**Table 1**). Therapy response was observed in 8/19 cases (42.1%), with one complete response (CR) and seven patients with partial response (PR, **Figures 1A, B**). Disease stabilization (SD) was observed in seven patients (36.8%), resulting in a disease control rate of 78.9% (**Figure 1A**). With a median follow-up of 29 months (range 1 to 56 months), median PFS was 24 months and median OS was 48 months.

Among *B2M*-wild type cancer patients, 7/15 (46.6%) showed therapy response, and 4/15 (26.6%) had SD (**Figure 1A**). Three patients showed progressive disease (PD, 20%), in one patient the first tumor assessment during ICB therapy is still pending.

Among 4 *B2M*-mutant cancer patients receiving ICB, only one patient (25% as opposed to 46.6% among *B2M*-wild type cancer patients, p>0.99), with initial disease stabilization, showed PR on subsequent imaging as best response, the other three patients had SD. No significant difference in PFS (median PFS: 19.5 *vs* 33.0 months, respectively, p=0.74, **Figure 1C**) or OS (median OS 39 months *vs*. not reached, respectively, p>0.99, **Figure 1D**) was observed between patients with *B2M*-mutant and *B2M*-wild type tumors. Also when using the start of palliative treatment as a reference time point, no significant influence of *B2M* mutation status on OS was observed (**Supplementary Table 1**).

## Discussion

### *B2M* Mutations and Prognosis

*B2M* mutations have been reported to be associated with prolonged survival for non-metastatic MSI cancer patients (8, 10, 15, 16). To the best of our knowledge, no systematic data exist about the survival of *B2M*-mutant stage IV MSI cancer patients. However, a previous study demonstrated that MSI GI cancer patients presenting with peritoneal metastases had a favorable baseline outcome compared to MSI GI cancer patients displaying a hematogenous metastatic pattern (17), notably without application of ICB therapy. We for the first time show that *B2M* mutation is associated with peritoneal metastasis in stage IV MSI GI cancer. Mutant *B2M* status significantly increased the odds of peritoneal metastasis, whereas the primary tumor site was not related to the metastatic pattern. *B2M* mutation therefore may represent a molecular marker of peritoneal metastasis and favorable baseline prognosis among stage IV MSI GI cancer.

What may be the mechanisms responsible for a favorable clinical course of *B2M*-mutant cancers in the absence of ICB? Previous studies have demonstrated that B2M loss limits the metastatic potential in MSI colorectal cancer (8, 15, 16) and other tumor types, such as uveal melanoma (18). Although the details are not yet fully understood, one hypothesis suggests that the lack of HLA class I antigens as ligands to the NK cell-inhibitory receptors renders *B2M*-mutant cells susceptible to NK cell-mediated elimination (18, 19). In addition, the association of tumor cells with platelets, a process relevant specifically for hematogenous metastases, seems to be disrupted by loss of HLA class I antigens (20). These factors may favor peritoneal metastasis and lower the likelihood of hematogenous spread. The hypothesis of *B2M*-mutant tumor cells being “trapped” in the peritoneum is supported by the metastatic patterns of *B2M*-mutant MSI cancers observed in our study.

### Clinical Implications

Our study for the first time provides clinical evidence that the biology and clinical course of metastatic *B2M*-mutant MSI GI cancers is substantially different from *B2M*-wild type MSI GI cancers. Considering that metastatic MSI cancers represent two distinct subgroups depending on *B2M* mutation status, the impact of *B2M* mutation status on response to ICB needs to be reconsidered. In particular, differences in baseline survival and prognosis need to be accounted for when analyzing the effectiveness of ICB in *B2M*-mutant and *B2M*-wild type GI cancers. The favorable outcome of *B2M*-mutant metastatic MSI cancer patients receiving ICB reported by Middha et al. (21) may be attributable to distinct baseline survival instead of representing actual treatment responses. Our data strongly encourage stratification of patients in clinical ICB studies according to *B2M* mutation status or peritoneal *vs*. hematogenous metastasis.

Previous studies also identified a potential impact of *KRAS*/*NRAS* mutations on ICB response (22). In our cohort, only 4 patients had colorectal cancer with *KRAS*/*NRAS* mutations, three of them (all *B2M*-wt) received ICB therapy and showed PR as best response. One patient had a colorectal cancer harboring both, *B2M* and *KRAS* mutations, but did not receive ICB. No significant correlation between *RAS* or *RAF* mutation status and presence or absence of *B2M* mutations was observed. Thus, although our study has limited power for analyzing the influence of *KRAS*/*NRAS* mutations on response to ICB therapy, no correlation between *BRAF* or *KRAS*/*NRAS* and the prevalence of *B2M* mutations could be observed.

**Figure 2:**
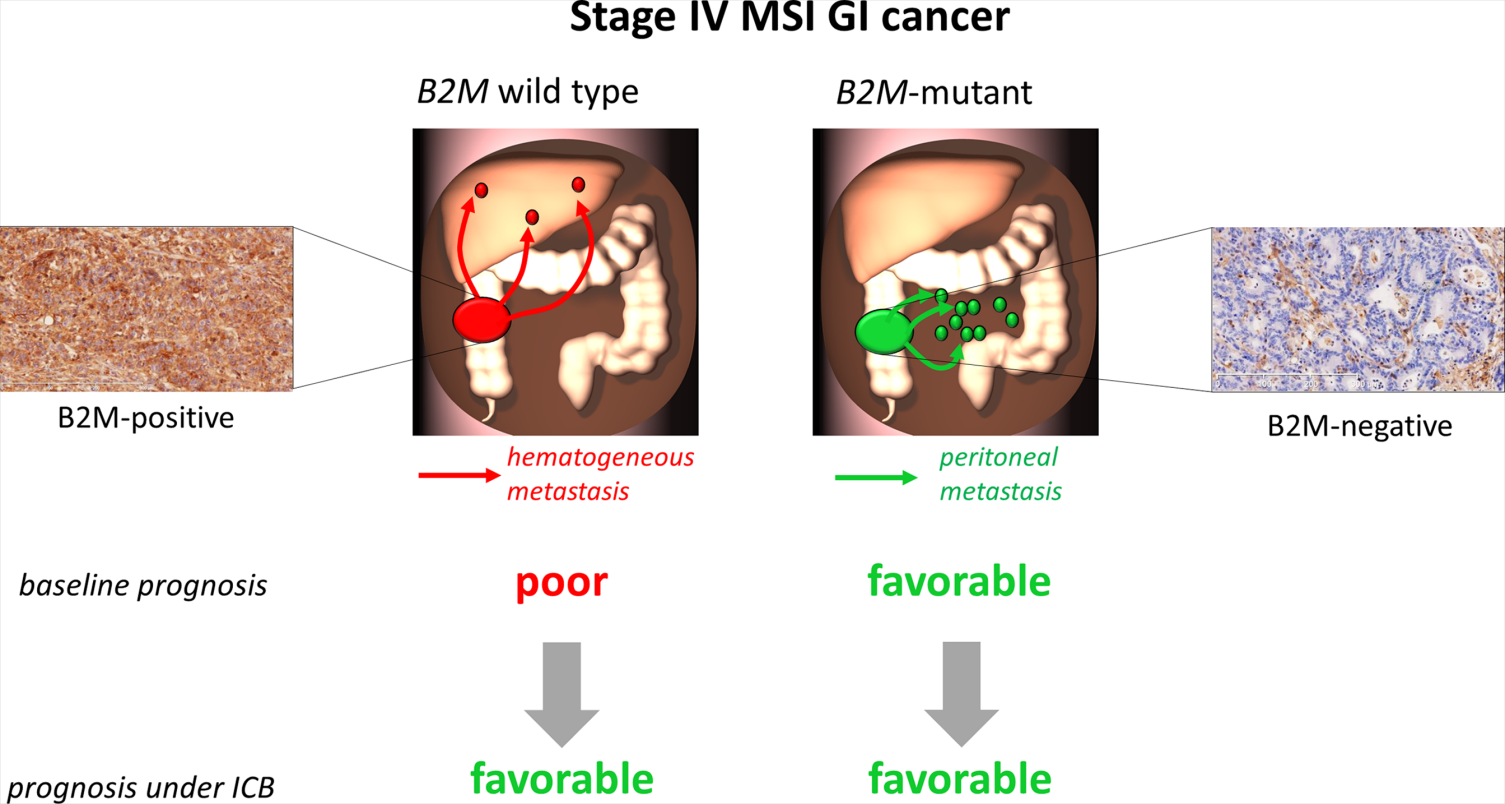
Hypothesis scheme. Patients with *B2M*-wild type MSI tumors and unimpaired B2M expression (left part of the figure) have a comparatively poor prognosis with high risk of hematogenous metastasis, thus these patients may benefit most from immune checkpoint blockade (ICB) therapy. In contrast, patients with *B2M*-mutant MSI tumors and loss of B2M expression (right part of the figure) due to biological characteristics of this tumor type rather present with a favorable prognosis and low risk of hematogenous metastasis. Thus, in these patients ICB therapy may not substantially improve the clinical picture.

The present study has strengths and limitations. The major strength is linking the previously described difference in baseline survival of stage IV MSI GI cancer patients to the *B2M* mutation status of the tumor and survival under ICB therapy. One limitation is that baseline survival data of *B2M*-mutant cancer patients are not available, as follow-up data were only retrieved for patients receiving ICB treatment. In addition, owing to the rarity of stage IV *B2M*-mutant MSI cancers, sample size of the present study is limited. However, the absence of *B2M* mutation status-dependent survival differences under ICB therapy is also compatible with the hypothesis that a biologically-determined favorable course of *B2M*-mutant tumors with peritoneal metastasis may leave limited room for further improvement under ICB therapy (**Figure 2**).

A study assessing the impact of B2M loss on ICB therapy in animal models demonstrated a major role of CD4-positive T cells in the immune response stimulated by combined anti-CTLA-4 and anti-PD-l therapy against tumors lacking MHC class I-associated antigen expression (23). Interestingly, this study also demonstrated limited effect of anti-PD-1 therapy alone on B2M-deficient tumors in mouse models and did not identify differences in OS or PFS in patients with tumors showing different B2M expression levels. This is in line with our findings showing no significant difference in PFS or OS between 15 *B2M*-wt and 4 *B2M*-mutant tumor patients receiving ICB therapy. Whether the patients with *B2M*-mutant tumors might benefit from combined ICB therapy approaches, remains to be confirmed in future human studies. In our study, only one patient with a *B2M*-mutant tumor and several metastases not restricted to peritoneal or lymphatic sites was treated with a combination of anti-CTLA-4 and anti-PD-l antibodies (ipilimumab and nivolumab) and demonstrated SD as best response.

Our study demonstrates that in stage IV GI MSI cancers peritoneal metastasis is closely associated with *B2M* mutations. The favorable prognosis associated with peritoneal metastasis in MSI cancer patients may mimic response to ICB. Future clinical trials are strongly encouraged to focus on this important clinical question. For that, prospective trials with larger patient cohorts recording metastatic patterns and *B2M* mutation status are required, which shall reveal whether the good survival of *B2M*-mutant M1 MSI cancer patients under ICB reflects the biology of the tumor cells, or indicates treatment response. This will ensure more tailored treatment selection for patients with metastasized MSI cancer and reduce treatment-related side effects in patients who may have no benefit from ICB. In addition, functional immunological studies contributing to understanding the mechanistic background of natural or ICB-induced immune response against tumors with impaired MHC class I expression are warranted.

Beyond the specific implications for *B2M*-mutant tumor patients and their response to ICB therapy, our study underlines the importance of better understanding the molecular characteristics of a tumor and their impact on patient’s prognosis when assessing the effect of certain therapies on patient’s survival.

## Data Availability Statement

The original contributions presented in the study are included in the article/**Supplementary Material**, further inquiries can be directed to the corresponding authors.

## Ethics Statement

The studies involving human participants were reviewed and approved by Ethics Committee of Medical Faculty, University Heidelberg. The patients/participants provided their written informed consent to participate in this study.

## Author Contributions

Conceptualization: EB, AA, GMH, and MK. Data Curation: EB, AA, KK, LB, PP, AB, JW, J-NW, and EHF. Formal Analysis: EB, AA, KK, LB, PP, EB, AB, JW, and J-NW. Funding Acquisition: MK, MKD, AA, and EB. Investigation: EB, AA, GMH, and MK. Methodology: EB, AA, GMH, and MK. Project Administration: GMH, and MK. Supervision: MKD, DJ, HB, EHF, GMH, and MK. Visualization: EB, AA, and MK. Writing – Original Draft: EB, AA, GMH, and MK. Writing – Review & Editing: all authors. All authors contributed to the article and approved the submitted version.

## Funding

This study was supported by Else Kröner-Fresenius Foundation (grant number 2018_A44, PI: MK), German Cancer Aid (grant number 70113455, Subroject PI: MK) and NCT Elevator Pitch (PIs: AA and EB).

## Conflict of Interest

GMH: Consulting or Advisory Role: Bristol-Myers Squibb, MSD Sharp & Dohme, EsoCap, Lilly; Honoraria: Servier, MSD Sharp & Dohme, Lilly; Research Funding: Nordic Pharma; Taiho Pharmaceutical, MSD Sharp & Dohme; Travel, Accommodations: Bristol-Myers Squibb; Lilly.

The remaining authors declare that the research was conducted in the absence of any commercial or financial relationships that could be construed as a potential conflict of interest.
