# Supplementary material for: Beta-2-microglobulin Mutations Are Linked to a Distinct Metastatic Pattern and a Favorable Outcome in Microsatellite-Unstable Stage IV Gastrointestinal Cancers
Source: Front Oncol. 2021 Jun 8;11:669774. doi: 10.3389/fonc.2021.669774 (PMC8219238; doi:10.3389/fonc.2021.669774)
Supplement: Supplementary file 1 [file Table_1.docx]

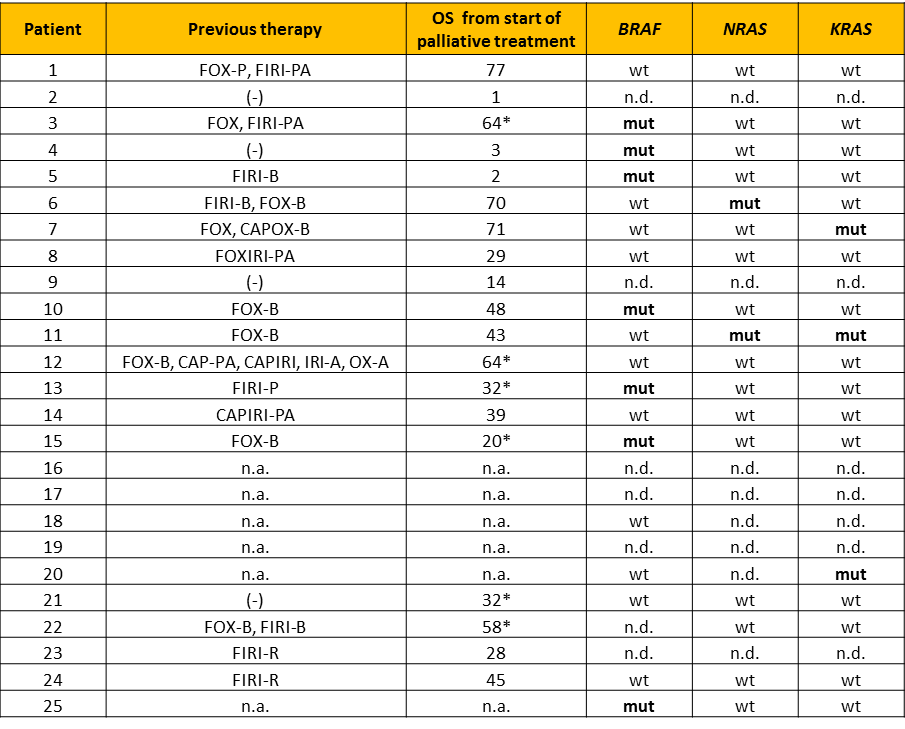


**Supplementary Table 1**. Additional clinical and molecular parameters of the described patient cohort, including data regarding previous therapy, OS after initiation of palliative treatment and RAF/RAS mutation status. Asterisks at OS value mark deceased patients. Abbreviations: FOX (FOLFOX), FIRI FOLFIRI), FOXIRI (FOLFOXIRI), CAPOX (Capecitabin/Oxaliplatin), CAPIRI (Capecitabin/Irinotecan), PA (Panitinumab), B (Bevacizumab), A (Aflibercept), R (Ramucirumab), p (Pembrolizumab), n/i (Nivolumab/Ipilimumab).
